# Fauna associated with Malayan filariasis transmission in Banyuasin, South Sumatra, Indonesia

**DOI:** 10.14202/vetworld.2021.1954-1959

**Published:** 2021-07-28

**Authors:** Budi Mulyaningsih, Sitti Rahmah Umniyati, Suwarno Hadisusanto, Erwin Edyansyah

**Affiliations:** 1 Department of Parasitology, Faculty of Medicine Public Health and Nursing, Universitas Gadjah Mada, Yogyakarta, Indonesia; 2 Department of Tropical Biology, Faculty of Biology, Universitas Gadjah Mada, Yogyakarta, Indonesia; 3 Postgraduate Program of Medicine, Faculty of Medicine Public Health and Nursing, Universitas Gadjah Mada, Yogyakarta, Indonesia

**Keywords:** *Brugia malayi*, lymphatic filariasis, *Mansonia uniformis*, South Sumatera, subperiodic nocturnal

## Abstract

**Background and Aim:**

*Brugia malayi* is known to be zoonotically important because it can be transmitted from animals (mammals and primates) to humans or from humans to humans through mosquito vectors. This study was conducted to explore the fauna associated with Malayan filariasis transmission in Sedang village, Suak Tapeh District, Banyuasin Regency, South Sumatra Province, Indonesia.

**Materials and Methods:**

A cross-sectional research design with an observational and analytical approach was applied in this study, and it was conducted in May 2018. Mosquitoes were collected twice using human bait both inside and outside the house from 6:00 p.m. to 6:00 a.m. The presence of competitors, predators, and reservoir hosts in the areas of five breeding habitats of *Mansonia* spp. was observed. The presence of microfilaria was confirmed under a microscope in night blood samples of inhabitants and cats. The presence of infective larvae (L3) of *B. malayi* was identified microscopically and based on the polymerase chain reaction method in female *Mansonia* mosquitoes.

**Results:**

A total of 12 mosquito species were found, among which *Mansonia uniformis* was the dominant mosquito, and the predominant competitor was *Mansonia annulifera*. Dragonflies, as predators were found in two breeding habitats and fish were found in one breeding habitat. The L3 of *B. malayi* were not identified in the mosquitoes, and the microfilariae of *B. malayi* were not found in the blood samples of inhabitants and cats.

**Conclusion:**

Although *Mansonia* mosquito population was abundant in Banyuasin Regency, the mosquito was not confirmed as an intermediate host of *B. malayi*, and the cat was not confirmed as a reservoir of *B. malayi* in the location.

## Introduction

Lymphatic filariasis is an infectious and chronic disease caused by *Wuchereria bancrofti*, *Brugia malayi*, and *Brugia timori* and is a major health problem in several tropical and subtropical countries. In 2004, an estimated 120 million people suffered from filariasis in 73 filariasis-endemic countries with an estimated 1.3 billion people in the filariasis transmission area [1,2]. This disease is widespread in almost all provinces in Indonesia. Nationally, during a period of 10-12 years, there was a significant increase in the number of cases and sufferers, from around 6500 cases in 2002 to 12,066 cases in 2011 and then to 14,932 cases in 2014, spreading in 418 districts/cities in 34 provinces. Epidemiologically, these data indicate that Indonesia is a high-risk area of contracting filariasis [3,4]. Lymphatic filariasis caused by *B. malayi* in South Sumatra Province is found in almost all districts, and until 2015, there were 232 patients with chronic filariasis. Banyuasin is one of the districts with numerous cases of filariasis, with several villages being included as high-endemic areas with Malayan filariasis [5]. In some endemic areas, the transmission of parasites is still occurring, which is indicated by the discovery of new clinical filariasis cases every year. The endemicity of an area reflects the interaction of several factors, that is, hosts (genetic and immunological), parasites (types, strains, and infective doses), mosquito vectors, and their environment [6,7]. Sedang village is an area with several rivers, paddy fields, and swamp forests, which are suitable *Mansonia* breeding habitats that can act as zoonotic Malayan filariasis vectors. *B. malayi* is known to be zoonotic because it can be transmitted from animals (mammals and primates) to humans or from humans to humans through mosquito vectors [8,9]. The existence of mosquitoes as a vector of filariasis transmission plays a key role in its spread that is associated with the environmental conditions and behavior of the locals. Some *Mansonia* spp. can be vectors of the zoonotic nocturnal subperiodic type of *B. malayi* [10,11]. According to some studies conducted in South Sumatra, the primary vectors of the zoonotic nocturnal subperiodic types of *B. malayi* are *Mansonia uniformis* and *Anopheles nigerrimus*; however, in Africa, *M. uniformis* and *Mansonia africana* are the vectors of *W. bancrofti* [12,13]. In Tanah Bumbu, South Kalimantan Province, Safitri [14] in 2012 found a mosquito species that was microscopically confirmed as a filariasis vector based on the presence of the infective larvae (L3) of *B. malayi* in *M. uniformis*, with an infective rate of 14.29%. It is also necessary to control the animal reservoirs that can be sources of filariasis infection. Studies conducted on cats (*Felis catus*) in Jambi reported that 33.3% (5 of 15) were infected with *B. malayi* [15,16]. Several species of mosquitoes belonging to four principal genera, namely, *Anopheles*, *Culex*, *Aedes*, and *Mansonia*, transmit filariasis. Their distribution, ecology, biology, and transmission potential vary significantly. Certain mosquito species occupy specific habitats, such as those with certain water quality requirements, namely, dissolved oxygen levels. For instance, the quality of life of *Culex quinquefasciatus* larvae will rapidly decrease at pH 9.4 [17].

It is important to understand the entomological aspects of the transmission of lymphatic filariasis because the transmission efficiency differs considerably according to the vector species [18]. The zoonotic nocturnal subperiodic type of *B. malayi* is transmitted by *Mansonia* spp. and can spread from animals to humans. One of the problems in the attempts to eliminate filariasis in Indonesia is the presence of animals that become reservoir hosts [12,19]. It is necessary to examine certain factors that play an important role in the spread of lymphatic filariasis to break the transmission chain. The best approach to break the transmission chain is case management (treatment of filariasis) and continuously observes filariasis vectors as the basis for vector eradication and eliminating mosquito breeding places [11,20].

The environment is highly influential on the transmission chain and the distribution of filariasis cases. The present study was conducted to evaluate the fauna associated with the transmission of Malayan filariasis, that is, the presence of *M. uniformis* as a vector, the presence of various species of mosquitoes as competitors, the predators that feed on mosquito larvae, and the presence of animals as reservoir hosts.

## Materials and Methods

### Ethical approval

This study has been approved by the Ethics Commission for Medical and Health Research, Faculty of Medicine, Public Health and Nursing, Universitas Gadjah Mada, Yogyakarta-Indonesia, with reference number: KE/FK/0389/EC/2018.

### Study design, period, and location

The study was conducted in May 2018. The research design was a cross-sectional survey and was conducted in Sedang Village, Suak Tapeh Subdistrict, Banyuasin District, South Sumatera Province, Indonesia.

### Mosquito collection and identification

Adult mosquitoes were captured using the human-landing method, which was performed by six people, with three people in the house and three people outside the house, for 12 h from 6:00 p.m. to 6:00 a.m., and it was performed in two consecutive nights. The captured mosquitoes were collected and then identified under a dissecting microscope [5,10]. The presence of L3 of *B. malayi* was identified microscopically and based on the polymerase chain reaction (PCR) method in female *Mansonia* mosquitoes.

### Observation of physical and biological factors

Ecosystem observations, including physical and biological factors, were performed in five places that were suspected of being *Mansonia* spp. breeding habitats. Physical factors included temperature (water, air, and soil), humidity, soil moisture, pH (water and soil), light intensity, clouds, and wind velocity. Biological factors included competitors and predators of *M. uniformis*, reservoir hosts of the nocturnal subperiodic type of *B. malayi*, and the abundance of plants found around the breeding place of *Mansonia* spp. The map coordinates and environmental conditions of each breeding habitat were observed and recorded.

### Blood sample collection and identification of microfilaria

The presence of microfilaria was confirmed under a microscope in night blood samples of inhabitants and cats. In this study, six cats were examined for blood microscopically and based on the PCR method to determine the presence of *B. malayi* L3. The PCR assay was performed using the forward primer HhaI (5'GCGCATAAATTCATCAGC-3') and the reverse primer HhaII (5'GCGCAAAACTTAATTACAAAAGC-3'). The PCR products were subjected to electrophoresis the results of which were positive if there were any bands with a size of 322 bp that formed [21].

## Results

A total of 426 mosquitoes were collected during the study, which consisted of five genera, that is, *Mansonia*, *Culex*, *Malaya*, *Armigeres*, and *Aedes*. *M. uniformis* constituted the maximum percentage (36.62% [156/426]) of mosquitoes collected using the landing method (Table-1). According to the study conducted by Pratiwi [22] from December 2016 to March 2017 using the indoor and outdoor human-landing collection method, there were 8239 mosquitoes consisting of 12 genera and 38 species. The genus *Mansonia* was found to be the most dominant in Sedang village. Further analysis revealed that environmental characteristics and the presence of aquatic plants played a key role in the diversity, abundance, and domination of mosquitoes. The existence and survival of mosquitoes are strongly influenced by the conditions of their breeding habitats. All vectors live according to local ecological conditions, including those living in brackish water at a certain level of salinity, whereas there are some vectors living in rice fields, along with clean water in the mountains and pools of water that can be exposed to sunlight [23]. Topographically, Sedang village is an area with several rivers, paddy fields, and swamp forests that are suitable for the breeding of *Mansonia* spp. Sedan village is also an area consisting of most of the agriculture, rubber, and oil palm plantations. In the present study, ecosystem observations, including physical and biological factors, were performed in five places that comprised the common types of breeding areas of *Mansonia* spp.

**Table-1:** The number and percentage of mosquitoes caught in Sedang Village, Suak Tapeh Subdistrict, Banyuasin District, South Sumatera Province, Indonesia.

Mosquito species	Number of mosquito	Percentage of mosquito
*Mansonia uniformis*	156	36.62
*Mansonia annulifera*	93	21.83
*Mansonia dives*	4	0.94
*Culex gelidus*	21	4.93
*Culex vishnui*	6	1.41
*Culex quinquefasciatus*	76	17.84
*Culex tritaeniorhynchus*	4	0.94
*Culex fuscosephalus*	25	5.87
*Malaya* spp*.*	9	2.12
*Armigeres subalbatus*	22	5.16
*Aedes aegypti*	9	2.11
*Aedes albopictus*	1	0.23
Total number of mosquitoes	426	100

Among the five breeding habitats in Sedang village, *Mansonia* spp. larvae were commonly found in breeding sites 2, 4, and 5; however, in breeding habitats 1 and 3, there were no *Mansonia* spp. larvae. The other insects found in breeding sites 2 and 5 were the adults and larval stages of the dragonfly, which can act as a predator as they feed on mosquito larvae. Fish were found in breeding habitat 5, which can also be a predator for mosquito larvae. Dragonfly and fish were found in the breeding habitats with numerous mosquito larvae because of the abundance of their food (mosquito larvae). Cats were found in every breeding habitat area and can act as reservoir hosts of the nocturnal subperiodic type of *B. malayi* (Table-2). We analyzed the physical factors in the five breeding habitats of *Mansonia* spp., including temperature, humidity, light, wind, and pH of water and soil (Table-3).

**Table-2:** Distribution of competitor, predator, and reservoir host in five *Mansonia* spp., breeding habitat areas in Sedang Village, Suak Tapeh Subdistrict, Banyuasin District, South Sumatra Province, Indonesia.

Observation	Breeding habitat of*Mansonia* spp.				

	1	2	3	4	5
Coordinate	S 2^0^ 49’ 39.5” E 104^0^25’ 34.7”	S 2^0^ 49’ 28.2” E 104^0^25’ 39.6”	S 2^0^ 46’53.5” E 104^0^29’ 35.8	S 2^0^ 39’ 35.2” E 104^0^25’ 19.4”	S 2^0^ 48’ 9” E 104^0^25’ 21.1”
*Mansonia* larvae	+	++++	−	+++	+
Culex larvae	+	−	−	−	-
Dragonfly	−	+	−	−	+
Fish	−	−	−	−	+
Cat	+	+	+	+	+

**Table-3:** Physical factors of five *Mansonia* spp. breeding habitats in Sedang Village, Suak Tapeh Subdistrict, Banyuasin District, South Sumatera Province, Indonesia.

Observation	Breeding habitat of*Mansonia* spp.				

	1	2	3	4	5
Water temperature	28°C	24°C	31°C	24°C	28°C
Air temperature	31°C	26°C	35°C	32°C	32°C
Soil temperature	29°C	25°C	30°C	29°C	30°C
Humidity	70%	86%	59%	86%	74%
Soil moisture	79%	90%	75%	90%	86%
pH of water	6.0	5.9	4.1	6	6.1
pH of soil	6.4	5.6	5.9	5.5	4.5
Light intensity	445	147	798	147	712
Claudy	70%	65%	90%	60%	55%
Wind velocity	0.095	0.096	0.096	0.96	1.87

In breeding habitat 2, the temperature of water, air, and soil was almost optimal (24-26°C) for insect growth and development, and in breeding habitat 2, the maximum number of *Mansoni*a spp. larvae was found. *Mansonia* spp. larvae were found on the roots of several water plants such as *Ipomoea aquatica*, *Eichhornia crassipes*, and *Limnocharis flava* in breeding sites 2, 4, and 5 in Sedang village. These water plants are highly essential for the growth and development of *Mansonia*, as they are extremely suitable for attaching the egg, larva, and pupa of *Mansonia* spp. The abundance of plants found in each of the breeding habitats of *Mansonia* spp. is shown in Table-4.

**Table-4:** The abundance of plants found in each breeding habitat of the *Mansonia* spp. in Sedang Village, Suak Tapeh Subdistrict, Banyuasin District, South Sumatra Province, Indonesia.

*Mansonia* spp. breeding habitat	Abundant plants in each *Mansonia* spp. breeding habitat
Breeding habitat 1	*Hymenachne amplexicaulis* (Rudge) Nees
	*Nypa fruticans*
	*Hymenachne* spp*.*
	*Mimosa pudica*
	*Ph Hymenahna yllanthus urinaria (Phyllanthus niruri)*
	*Azolla pinnata*
	*Eichhornia crassipes*
Breeding habitat 2	*Ipomoea aquatic*
	*Hymenachne amplexicaulis* (Rudge) Nees
	*Ileusin tricocephala*
	*Hymenachne* spp.
	*Ageratum conyzoides*
	*Hevea brasiliensis*
	*Nypa fruticans*
Breeding habitat 3	*Sonneratia caseolaris*
	*Rhizophora*
	*Nypa fruticans*
	*Nymphaea* (water lily)
	*Ileusin tricocephala*
	*Azolla pinnata*
	*Elaeis* (oil palm)
Breeding habitat 4	*Azolla pinnata*
	*Eichhornia crassipes*
	*Limnocharis flava*
	*Hymenachne spp.*
	*Hymenachne amplexicaulis* (Rudge) Nees
	*Tracheophyta*
	*Hevea brasiliensis*
Breeding habitat 5	*Elaeis* (oil palm)
	*Nymphaea* (Water lily)
	*Melaleuca leucadendra syn. M. leucadendron*
	*Melastoma*
	*Hymenachne amplexicaulis* (Rudge) Nees
	*Hevea brasiliensis*
	*Hymenachne* spp*.*

We found negative results in the microscopic examination and the molecular PCR method conducted to identify the L3 of *B. malayi* in female *Mansonia* mosquitoes. This finding indicates that filarial larvae were not detected in all the captured mosquito species.

## Discussion

Epidemiologically, the transmission of lymphatic filariasis involves several complex factors, such as the presence of lymphatic filarial worms as agents of disease, humans as hosts, adult mosquitoes as vectors, and physical, biological, and social environmental factors. The environment is highly influential on the distribution and transmission chain of filariasis cases. The biological environment involved in the transmission chain of filariasis includes the presence of aquatic plants as a place of *Mansonia* spp. growth, the presence of other mosquitoes as competitors, the presence of other insects or animals as predators, and the presence of animals as reservoir hosts.

Although the population of *Mansonia* mosquitoes was found to be abundant in this study, no *B. malayi* L3 were identified. Therefore, *Mansonia* mosquitoes were not confirmed as an intermediate host of *B. malayi* in this study. The microfilariae of *B. malayi* were also not found in cat blood samples in this study; therefore, cats were not confirmed as the reservoir of *B. malayi* in this location. Similarly, a study conducted in 2006 in Sungai Rengit village, Banyuasin Regency, South Sumatra [23], and a study conducted in 2011 in Muara Padang village, Banyuasin District, South Sumatra [24], reported that *B. malayi* L3 was not identified in the mosquitoes. Conversely, a study conducted in Narathiwat Province, South Thailand [25], and another study conducted in East Kalimantan [26] reported the presence of *B. malayi* microfilariae in cat blood samples.

The development of insects, including mosquitoes, is influenced by two factors, that is, the internal factors from the insects themselves and the external factors that are present in the surrounding environment where the insects live. High or low insect populations at any given time are influenced by these two factors [27]. All vectors live according to local ecological conditions. The conditions of mosquito breeding habitats are largely determined by the existing environmental conditions, such as vegetation, predators, temperature, humidity, and rainfall. A strong positive correlation exists between the abundance of mosquitoes, physical factors, and chemical factors in their breeding places [28].

Geographically, the mosquito is a cosmopolitan insect with a widespread distribution in the tropical and subtropical regions, and the changes in the environment influence the activity of the insect, which has an impact on its diversity and distribution [29]. Mosquitoes grow normally at optimum temperatures between 25°C and 27°C. Low temperatures inhibit larval growth, and high temperatures kill larvae [28]. The results of this study also demonstrated the presence of *Mansonia* spp. in the breeding habitats with optimum temperature. Biological environment includes all the living things that are found around humans, that is, flora and fauna, which can act as a chain of filariasis transmission. *Mansonia* spp. are associated with swamps and large rivers on the edge of the forest or in the forest. Larvae and pupae with their siphon are attached to the roots or branches of aquatic plants such as *E. crassipes*, *Nymphaea* (water lily), and *I. aquatica* [11]. The vegetation in mosquito breeding habitats, including mangroves, mosses, algae, and various other plants, can affect the life of larvae as they can block sunlight or protect against other living creatures. Aquatic plants affect the breeding and survival potential of *Mansonia* spp. In *Pistia* *stratiotes* and *E. crassipes*, the aerenchyma (a soft plant tissue containing air spaces, found especially in several aquatic plants) is larger in size than that in *Mimosa pudica* and *Azolla pinnata* and can store more oxygen. The root tissues of *M. pudica* and *A. piñata* are more rigid and can inhibit perforation by the siphon larvae of *Mansonia* spp. The root structure of *P. stratiotes* and *E. crassipes* can reduce the effectiveness of natural predators from mosquito larvae in ponds. *A. pinnata* is believed to have secondary metabolites that are dangerous to *Mansonia* spp. larvae [30].

Endemic areas of *B. malayi* are generally found around rivers, forests, swamps, along rivers, or other water bodies overgrown with aquatic plants. Areas with different flora have different disease patterns. Swampy or muddy environment and bushes around houses are suitable breeding habitats for the Malayan filariasis vector (*Mansonia* spp.), and there exists a relationship between the presence of aquatic plants and the incidence of filariasis. Therefore, people living in endemic areas in houses with aquatic plants could have a risk of transmission of filariasis [31].

## Conclusion

This study found 12 mosquito species, among which *M. uniformis* was the dominant mosquito, and the predominant competitor was *Mansonia annulifera*. The study results also indicated the presence of dragonflies as predators in two breeding sites and the presence of fish in one breeding habitat. Furthermore, despite the abundant population of *Mansonia* mosquitoes in Banyuasin Regency, the mosquito was not confirmed as an intermediate host of *B. malayi*, and the cat was also not confirmed as a reservoir of *B. malayi* in the location.

## Authors’ Contributions

BM: Designed the study. SRU and SH: Conducted the field survey. EE: Collected mosquito sample. All authors drafted, revised, read, and approved the final manuscript.
